# Development and Evaluation of an Innovative Web-Based Training, Learning, and Sharing Platform for Social Workers (Hong Kong Jockey Club SMART Family-Link Project): Mixed Methods Evaluation Study

**DOI:** 10.2196/32894

**Published:** 2022-04-28

**Authors:** Michelle Man Tung Suen, Agnes Yuen Kwan Lai, Man Ping Wang, Daniel Sai Yin Ho, Tai Hing Lam

**Affiliations:** 1 School of Public Health The University of Hong Kong Hong Kong China (Hong Kong); 2 School of Nursing The University of Hong Kong Hong Kong China (Hong Kong)

**Keywords:** web-based, learning platform, capacity building, social work practice, ICT, social work, professional development, information and communication technology, Google Analytics, family services, mobile phone

## Abstract

**Background:**

Information and communication technology (ICT) use may enhance social work practice and continuous professional development. Under the Hong Kong Jockey Club SMART Family-Link Project, we developed an innovative web-based training, learning, and sharing platform (i-TLS) to support not only ICT and other learning needs of Hong Kong social workers but also their practice.

**Objective:**

We developed i-TLS with 3 major components (i-Training, i-Learning, and i-Sharing) and assessed its acceptability and impact on facilitating ICT use in family services.

**Methods:**

We described the i-TLS development based on a 4-phase model and evaluated i-TLS using the platform database, Google Analytics, a self-administered survey, and individual phone interviews 1 year after launching.

**Results:**

i-TLS was launched in 12 nongovernmental organizations on July 1, 2019. The COVID-19 outbreak in December 2019 limited face-to-face services, which galvanized digital transformation in social work practice. By July 31, 2020, 313 social workers had registered with i-TLS. Approximately 79.6% (249/313) of users accessed i-TLS at least once in the past 28 days, averaging 3.2 (SD 1.35) platform visits per day and viewing 4.8 (SD 1.42) pages per visit. i-Training provided 41 mini-modules on applying ICT to family services, with 730 enrollments. Approximately 70% (511/730) of users completed the mini-modules and obtained digital mini-certificates. i-Learning provided 112 items of learning resources centered on ICT use in family services, with nearly 4000 page views. i-Sharing had 25 discussion threads with 59 posts. Approximately 53.7% (168/313) of users completed the 1-year evaluation survey, including 7.1% (12/168) who were phone interviewed. The mean i-TLS satisfaction score (out of 10) increased from light (4.99, SD 1.54) to occasional (6.15, SD 1.34) and frequent (6.31, SD 2.29) users. Frequent users showed higher scores (out of 10) than light users for an increase in knowledge (5.84, SD 1.34 vs 4.09, SD 1.74; *P*<.001), self-efficacy (5.23, SD 1.92 vs 3.96, SD 1.77; *P*=.02), and knowledge application (6.46, SD 1.33 vs 1.91, SD 1.40; *P*<.001). Interviewees reported increased ICT use in services and considered i-TLS an acceptable and supportive tool for learning and practice, especially during the pandemic.

**Conclusions:**

i-TLS is acceptable to social workers and enhances their learning and use of ICT in family services. This was achieved through access to self-directed and collaborative learning and sharing of experiences within their practice. Further research on enhancing web-based platforms is needed to expand participation and capacity building among social workers and other health and social care professionals.

## Introduction

### Background

Widespread use of information and communication technology (ICT) has revolutionized human communications and the way social work practitioners and clients connect [1]. This has fueled the demand for ICT use in social work practice to improve service delivery and benefit society [2]. Social workers can choose from a wide range of ICT service delivery options, such as web-based counseling, self-help web-based interventions, social networking, email, and SMS text messaging [3], that have enhanced their availability and interaction with individual clients and specific groups and achieved better intervention outcomes [4,5].

Traditional face-to-face training with a fixed schedule and venue has restricted its reach and social workers’ opportunities of learning. Skill and knowledge exchanges are limited and often confined to colleagues within the same center or organization. Before the COVID-19 pandemic, despite the potential benefits of ICT use, many social workers were hesitant to adopt ICT at work [6]. This may relate to the importance of in-person relationships in social work or the constraints on resources and knowledge in digital practice. Limited digital competence may hinder social workers from realizing the potential of ICT to support their practice. With limited exposure to a technology-rich environment, it will be more difficult for social workers to integrate technology into service effectively [4,7]. This in-person learning approach is essential but not sufficient as a method to meet the needs of the fast-growing digital practice.

Conducting traditional teaching or on-site training can be challenging in certain situations, such as a pandemic [8,9]. The COVID-19 pandemic has significantly changed the context of ICT use in social work practice and learning. Owing to the unprecedented suspension of all nonessential in-person services, social workers are forced to rely on ICTs for most practice and communication with clients. Without preparation, an emerging and instant difficulty for social workers is how to transit and effectively use ICT to maintain service [10,11]. This urgent situation necessitates social workers to adopt more asynchronous and shared approaches to learning. They need a space where they can quickly access updated information and share a body of knowledge, tools, and ideas and learn from each other about how to improve web-based services and outcomes. During these challenging times, a web-based learning platform can offer timely support in providing essential learning resources at social workers’ fingertips and leveraging technology to create a learning community that can benefit practice in the long term. Our search of PubMed and Social Care Online, using keywords such as *social work* and *online learning* or *digital learning* or *learning platform*, up to July 27, 2021, found only 1 study [12] on creating a platform for social work clinical practice in a health care setting; however, none were found on the development and evaluation of a web-based platform for social workers to learn about ICT use in family services.

The Hong Kong Jockey Club SMART Family-Link Project, funded by The Hong Kong Jockey Club Charities Trust since 2018, is a collaboration between the School of Public Health and the Technology-Enriched Learning Initiative of the University of Hong Kong (HKU) and 26 Integrated Family Service Centers and Integrated Services Centers operated by 12 nongovernmental organizations (NGOs) in Hong Kong. The goal of the project is to develop an advanced family service delivery system through the cost-effective use of ICT to support and enhance service delivery so as to promote family health, functioning, and well-being. Before this project, ICT use was minimal across the family service centers [13].

Apart from public education to promote family well-being by the HKU project team, the project includes design and development in close collaboration with all Integrated Family Service Centers or Integrated Services Centers of an i-Connect system and an i-Action component for promoting the use of ICT in preventive family service delivery. The i-Connect system includes a client information system, casework, group and program management, assessment tools, and statistical reports. It helps to reduce administrative work, facilitate the early identification of at-risk cases, and perform data analysis across centers and NGOs. i-Action promotes the use of ICT in program design and planning in services by cocreating a wide range of ICT tools with NGO partners. The tools include e-message portals, mobile apps, and family-friendly games to engage target families and further enhance service reach. As part of the project, an innovative web-based training, learning, and sharing platform (i-TLS) was developed, in parallel, to supplement the traditional learning of social workers regarding ICT use and facilitate its clinical and community applications. It is a multicomponent and web-based platform comprising social work and ICT-specific information covering tips and training in applying ICT to family services from counseling, program design, and implementation to evaluation.

**Figure 1:**
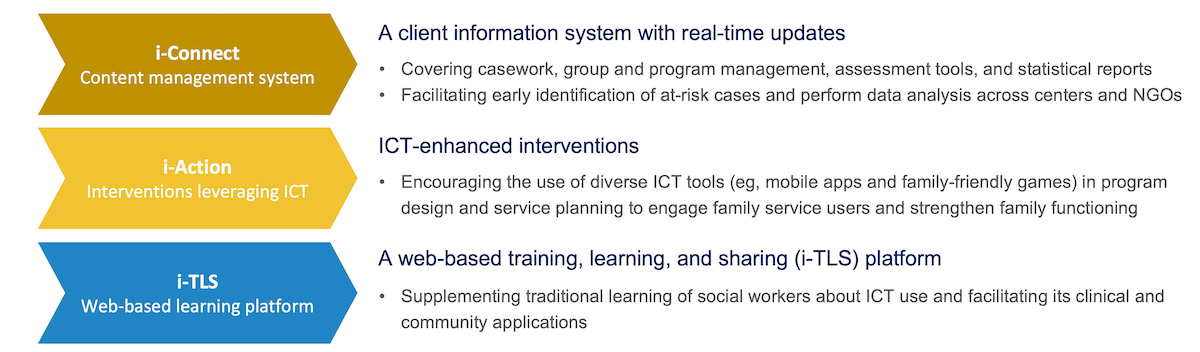
Overview of the three project components (i-Connect, i-Action, and i-TLS). ICT: information and communication technology; NGO: nongovernmental organization.

The i-Connect system was launched in May 2020, and i-Action had its first ICT-enhanced program implemented in early July 2018. Throughout the implementation process, i-TLS provided additional training support and important information tied to i-Connect and i-Action. A summary of the project components—i-Connect, i-Action, and i-TLS—is presented in Figure 1, and the interface of the i-TLS components is presented in Multimedia Appendix 1.

### Objectives

More than a website for information sharing, i-TLS encourages the collective wisdom of large groups of social workers to address the challenging conditions of the COVID-19 pandemic and facilitate ICT-enhanced services. Users are expected to improve their knowledge and skills in using digital tools in ways that benefit the values of the social work profession, enhancing service quality, efficiency, and experience. The aim of this paper is to provide an overview of the i-TLS design and development for social workers to learn about ICT use in family services and evaluate its acceptability and impact in its first year using both quantitative and qualitative methods.

## Methods

### i-TLS Design and Development

**Figure 2:**
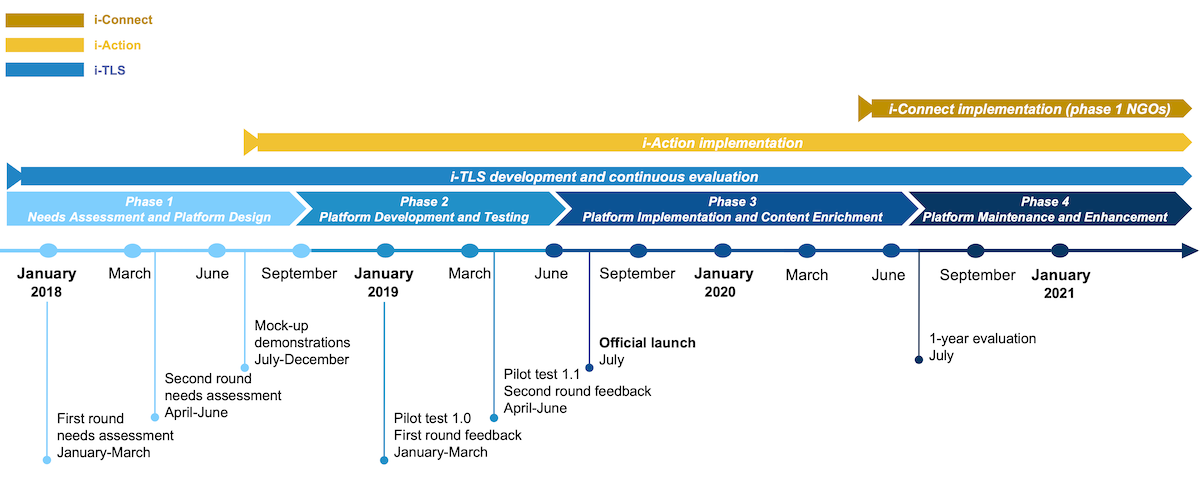
The development of the innovative web-based training, learning, and sharing platform (i-TLS) in 4 phases and the other 2 project components. NGO: nongovernmental organization.

By integrating the principles of asynchronous and collaborative learning, i-TLS was developed based on a 4-phase model with a continuous evaluation that spanned the 4 phases. Figure 2 shows the development of i-TLS and the other 2 project components.

#### Phase 1: Needs Assessment and Platform Design

##### Overview

**Figure 3:**
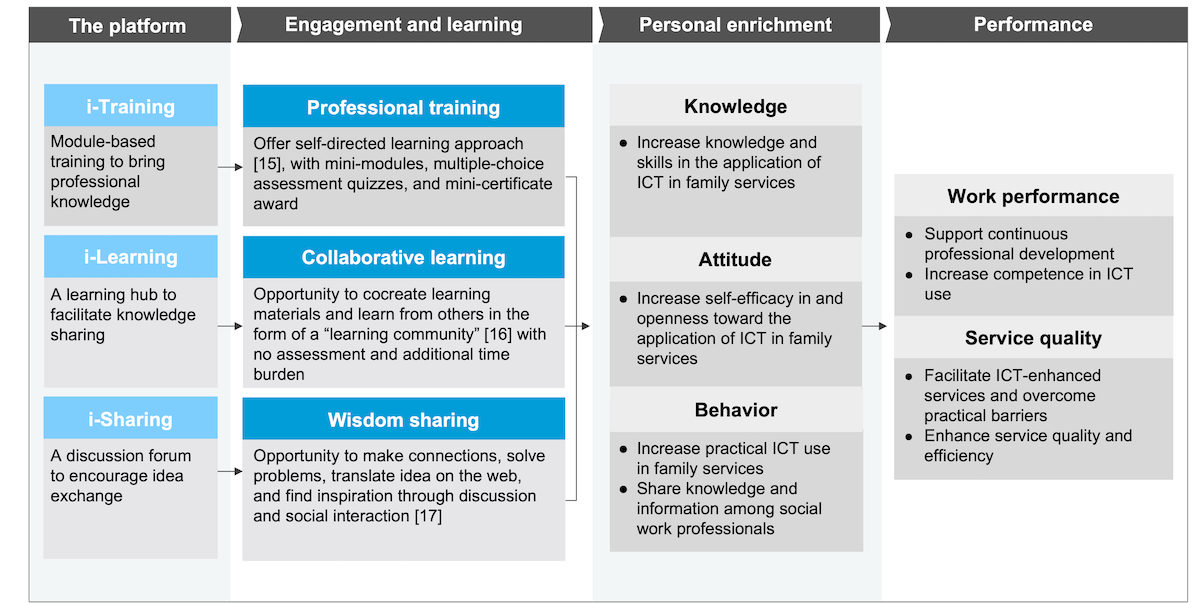
Overview of the innovative web-based training, learning, and sharing platform (i-TLS) components [15-17]. ICT: information and communication technology.

From January 2018 to June 2018, 16 face-to-face focus groups were conducted with 12 NGO partners to understand their learning needs, attitudes, and barriers to ICT use. Some of them participated twice as they have different centers across service boundaries. Overall, 108 social workers, including 28 (25.9%) supervisors and 80 (74.1%) frontline workers, participated. Approximately 61.1% (66/108) of them were women, and the median number of participants per group was 5 (range 3-21). Qualitative methodologies are optimal when the researcher wants to examine participants’ views and experiences [14]. Semistructured interviews with open questions allow for flexibility and the discovery of more in-depth information that is important to participants and researchers. The information was analyzed to help design the i-TLS content and format. The design of the individual components (i-Training, i-Learning, and i-Sharing) and their theoretical backgrounds are shown in Figure 3 [15-17].

##### i-Training

i-Training adopts a self-directed learning approach [15], which includes self-monitoring and self-evaluation mini-modules designed by a multidisciplinary team of health, ICT, and social work professionals for materials that are considered to be most important, including 4 categories: data privacy and system security, ICT use in practice, program evaluation and ICT tools. On the basis of the concept of cognitive load theory [18-20], the contents were designed as mini-modules with multiple-choice assessment quizzes that can be completed in approximately 15 minutes to prevent information overload. It has been suggested that minimizing the extraneous load enables more working memory resources to be available that facilitate learning [20,21]. Social workers who completed a mini-module with a score of ≥50% in a quiz were awarded a digital mini-certificate. They could re-enroll or retake the module anytime.

##### i-Learning

i-Learning is a learning hub with no assessment. It adopts a flexible and collaborative approach that encourages social workers from different centers to join a colearning circle with less time burden than traditional methods. Social workers cocreated learning materials and tip packs, including sharing of successes and lessons learned in ICT-enhanced practice, with the HKU project team and shared any resources that are useful for social services. The contents are shareable, practical, and high-yield educational, benefiting social workers in the form of communities of practice through sharing their common concerns, learning from each other’s experiences and growing in their practice [16]. We aim to support such communities of practices to enhance social workers' training and services that are frequently absent in the bustling clinical learning environment.

##### i-Sharing

i-Sharing is a sharing and discussion forum through which social workers can interact and exchange ideas on topics related to their professional interests or project information. They can also post questions, remarks, and comments on the training and learning resources to support ICT use in social services and professional development in social services and professional development. Through this process, users have the opportunity to make connections, solve problems, and find inspiration from others, which are crucial factors in learning [17]. Their feedback also guided us in platform improvements and planning.

#### Phase 2: Platform Development and Testing

We consolidated the learning content and multimedia tools. A series of digital learning resources with videos, graphics, and quizzes was created for a mock-up. Showing a video can provide easier understanding than a text description of complex ideas or procedures [22], whereas these diverse formats of web-based learning materials with quizzes may increase users’ engagement in web-based learning use. Every feature of the platform was tested with all participating users in 2 pilot tests from January 2019 to June 2019 to ensure the usability of the platform and identify key challenges.

#### Phase 3: Platform Implementation and Content Enrichment

On the basis of the users’ feedback, we conducted a thorough review of the platform and made the necessary adjustments before implementation. i-TLS was officially launched for all supervisors and frontline workers of 12 NGO partners on July 1, 2019. In parallel, we continued to enrich the learning content and facilitate cocreation with NGO partners. A series of training and learning resources, such as practical tips, prototypes, manuals, and video tutorials, was uploaded to i-Training and i-Learning on a regular basis.

#### Phase 4: Platform Maintenance and Enhancement

To enhance the users’ experience, we worked with web developers to conduct platform maintenance. All information from the continuous evaluation was analyzed and incorporated into a new platform design to enhance navigation, display, layout, and user engagement.

### Continuous Evaluation

As the HKU project team interacted with the NGO partners, their feedback and needs emerged through both formal inquiries and informal conversations. Collective platform use and activity data were reported to stakeholders each month to evaluate the development and progress of the platform. Approximately 1 year after launching i-TLS, we used the platform database, Google Analytics, a self-administered anonymous survey questionnaire, and individual phone interviews to assess its acceptability and impact in facilitating social workers’ learning and ICT-enhanced family services.

### Process Evaluation

#### Acceptability: Use

We assessed acceptability based on the number of registered and active users, platform use, and reactions to the platform. Registered users were those who were registered with i-TLS during the evaluation period (from July 1, 2019, to July 31, 2020). Active users were those who accessed i-TLS at least once in the past 28 days. Platform use included frequency of use and platform activity. Frequency of use was assessed using Google Analytics data on the duration and number of pages per visit and the following survey item: “In the past 3 months, to what extent did you use the platform?” Responses were given on an 11-point Likert scale ranging from *0=never* to *10=every day* and were analyzed as light users (score of 0-3), occasional users (score of 4-5), and frequent users (score of 6-10). Platform activity was directly assessed using the platform database on the number of mini-module completions and discussion posts, as well as Google Analytics data on the number of page views.

#### Acceptability: Reaction to Platform

The users’ reactions to i-TLS were assessed using 2 survey items and phone interviews to cover their overall satisfaction with i-TLS and their likelihood of recommending it to others. Survey respondents were asked, “In general, how satisfied have you been with i-TLS?” and “How likely will you recommend the platform to other colleagues?” Responses were given from *0=very dissatisfied* to *10=very satisfied* using a dichotomous scale—*yes* and *no*—respectively.

### Outcome Evaluation

#### Impact on Learning

We assessed the impact of i-TLS on learning and behavior. Learning was assessed using 2 survey items: “To what extent did you gain knowledge of applying ICT to family services?” and “To what extent did you feel confident in applying ICT to family services?” Responses ranged from *0=not at all* to *10=very highly increased*.

#### Impact on Behavior

We assessed the impact of i-TLS on learning using 2 items on the frequency of knowledge application and sharing: “In the past 3 months, how often did you apply the acquired knowledge to family services?” and “In the past 3 months, how often did you share the content of the platform to your colleagues in family services?” Responses ranged from *0=not at all* to *10=every day*.

We also conducted semistructured individual phone interviews with both supervisors and frontline workers to explore the usefulness of i-TLS for learning and practice and the practical barriers to ICT use in service.

### Ethics Approval

Ethical approval was granted by the Institutional Review Board of HKU and Hospital Authority Hong Kong West Cluster (UW19-449) on July 11, 2019. This study was also registered at the National Institutes of Health (NCT04034420) on July 29, 2019. All participants provided web-based written informed consent at the beginning of the study.

### Data Collection

From July 2020 to August 2020, we invited all 313 users (n=23, 7.3% supervisors and n=290, 92.7% frontline workers) to complete a self-administered anonymous survey questionnaire via email. A total of 168 users (n=18, 10.7% supervisors and n=150, 89.3% frontline workers) responded to our survey, and the 12 users (n=3, 25% supervisors and n=9, 75% frontline workers) who indicated willingness were randomly selected to participate in individual phone interviews from August 2020 to September 2020. The first author (MTMS) conducted and recorded all interviews, each lasting approximately 10 to 20 minutes with a mean of 15 minutes.

### Data Analysis

Quantitative analyses were performed using SPSS Statistics (version 25.0; IBM Corp). One-way ANOVA followed by post hoc tests was used to compare the means between subgroups (light users, occasional users, and frequent users). All significance tests were 2-sided, with *P*<.05 indicating statistical significance. Effect size (Cohen *d*) was calculated with values of 0.2, 0.5, and 0.8 indicating small, medium, and large effects, respectively [23].

Phone interview data were analyzed using NVivo (version 12; QSR International) and thematic analysis [24]. Thematic analysis is a method of analyzing qualitative data that provides a rich account of the data collected [24]. In this study, it was used as a realist method to report the experiences of users. Transcripts were coded by the first author (MTMS) and a research assistant of the project, with guidance from an experienced researcher (AYKL). Themes were coded deductively based on the outcome assessment at the manifest level to provide personal insights and practical examples for social work professionals and researchers. Coding was conducted systematically, and the identified themes were reviewed and refined continually to ensure that they reflected the original meaning of the data set.

The consistency of an independent coder attributing themes is often used as a method of checking the reliability in qualitative methods [25]. Coding was conducted systematically, and the identified themes were reviewed by the experience researcher (AYKL) and refined continually to ensure that they reflected the original meaning of the data set. In total, 2 independent researchers were given the codebooks and interview excerpts and asked to code the excerpts using the themes to check the reliability of the themes.

## Results

### Survey Respondent Characteristics

Table 1 shows that 72% (121/168) of respondents were women, 49.4% (83/168) were aged 30 to 39 years, and 84.5% (142/168) had a degree or higher education. Approximately 89.3% (150/168) were frontline workers, and 52.4% (88/168) had at least 9 years of social work experience. Approximately 59.5% (100/168) had registered with the platform for <1 year.

**Table 1:** Characteristics of the survey respondents (N=168).

Characteristic			Survey respondents, n (%)
**Sex**			
	Male	47 (28)	
	Female	121 (72)	
**Age group (years)**			
	18-29	43 (25.6)	
	30-39	83 (49.4)	
	40-49	31 (18.5)	
	≥50	11 (6.5)	
**Education level**			
	Diploma, subdegree, or below	26 (15.5)	
	Degree or above	142 (84.5)	
**Post**			
	Frontline worker	150 (89.3)	
	Supervisor	18 (10.7)	
**Social work experience (years)**			
	0-2	35 (20.8)	
	3-8	45 (26.8)	
	9-15	88 (52.4)	
**Registration duration (years)**			
	<1	100 (59.5)	
	≥1	68 (40.5)	

### Overview of Qualitative Themes

The phone interview data provided a deeper understanding of user views and experiences using i-TLS and ICT in social services. Textbox 1 shows the 5 key subthemes that emerged from our data analysis.

**Overview of qualitative themes.**

**Themes and subthemes**

- Reactions to innovative web-based training, learning, and sharing platform
  Enabled access to self-directed learning and resources
- Impact on learning
  Enhanced memorization and application of information and communication technology (ICT) in services
  Facilitated knowledge transfer through collaborative learning
- Impact on behavior
  Increased openness and ICT use
  Barriers to effective ICT-enhanced practice

### Acceptability

#### Registered and Active Users

**Figure 4:**
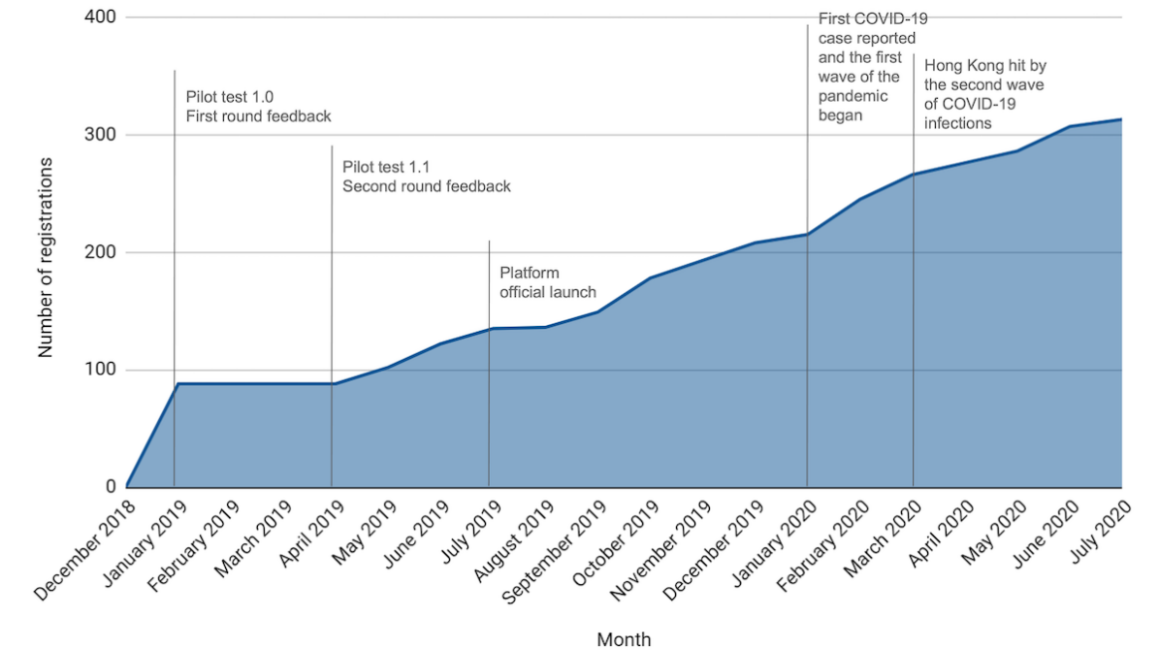
Registration trend since the platform pilot launch as of July 31, 2020.

Figure 4 shows that i-TLS was launched to 135 initial users on July 1, 2019. The number of users has substantially grown thereafter. During the first wave of the COVID-19 pandemic, the number of users rose from 215 in January 2020 to 245 (14%) in February 2020. By July 31, 2020, 313 users (n=23, 7.3% supervisors and n=290, 92.7% frontline workers) from 12 NGO partners had registered. Six NGO partners reached a 100% registration rate the registration rates of the remaining 6 NGO partners ranged from 5.2% to 57.1% by July 31, 2020. Although the number of users doubled in a year, Google Analytics showed that 79.6% (249/313) of the users were active in the past 28 days during the evaluation period.

#### Platform Use

##### Overview

The average duration of the visits was 4.3 minutes. The average number of visits per user per day was 3.2, and the average number of pages per visit was 4.8. Of the 168 survey respondents, 76 (45.2%) were light users, 79 (47%) were occasional users, and 13 (7.7%) were frequent users. On the basis of the platform database and Google Analytics data, the most commonly visited resources across i-Training, i-Learning, and i-Sharing were related to ICT use in practice.

##### i-Training

A total of 41 mini-modules in 4 categories were created and uploaded to i-Training from July 1, 2019, to July 31, 2020. Table 2 shows a total of 730 enrollments in the mini-modules, with 70% (511/730) awarded digital mini-certificates. Training on data privacy and system security had the highest average number of enrollments, with 71.5% (271/379) completion. Within each of the 4 categories, mini-modules *Data Best Practice*, *The Application of E-messaging Intervention in Services*, *The BACK* Model, and *i-Connect System—Group/Program Enrolment* were the most popular, with 90.9% (50/55), 80% (28/35), 78% (18/23), and 75% (6/8) completed, respectively.

**Table 2:** Number of enrollments in and completions of the 41 mini-modules in i-Training.

Category	Minimodules (N=41), n (%)	Enrollments (N=730), n (%)	Enrollments per mini-module, mean (SD)	Completions^a^ (N=511), n (%)	Completions^a^, mean (SD)	Completion rate (%)
Data privacy and system security	13 (32.3)	379 (51.9)	29.2 (13.1)	271 (53)	20.8 (13)	71.5
ICT^b^ use in practice	5 (12.2)	127 (17.4)	25.4 (18)	94 (18.4)	18.8 (16)	74
Program evaluation	3 (6.8)	72 (9.9)	24.0 (1)	54 (10.6)	18.0 (0.5)	75
ICT tools	20 (49.4)	152 (20.8)	7.6 (4.9)	92 (18)	4.6 (3)	60.5

^a^Including all who completed the post–mini-module quizzes with an overall score of ≥50% and were awarded mini-certificates.

^b^ICT: information and communication technology.

##### i-Learning

**Figure 5:**
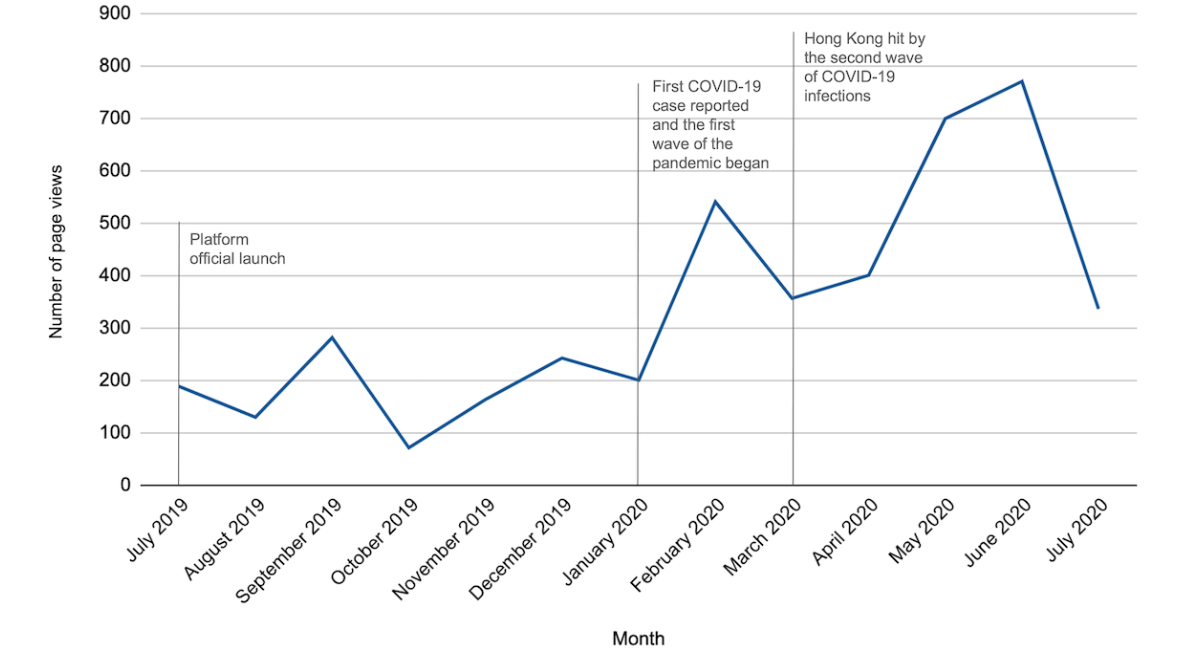
Number of page views in i-Learning by month from July 1, 2019, to July 31, 2020.

A total of 112 items of learning resources were created and uploaded to i-Learning from July 1, 2019, to July 31, 2020, with 4388 page views recorded from Google Analytics. Table 3 shows that the item with the highest average number of page views was experience sharing of ICT use in practice, followed by ICT tools and games. Figure 5 shows the number of page views by month for the period from July 1, 2019, to July 31, 2020. The number of page views maintained steadily between 100 and 300 in the first 6 months but increased sharply from 201 in January 2020 to 541 following the first COVID-19 case reported. The number of page views slightly dropped in March 2020 but rebounded and surged to 771 in June 2020 after the second wave of the COVID-19 pandemic hit in mid-March.

**Table 3:** Number of items and page views in i-Learning.

Category	Items (N=112), n (%)	Page views (N=4388), n (%)	Page views per item, mean (SD)
Experience sharing of ICT^a^ use in practice	8 (7.13)	696 (15.86)	87.0 (27.7)
ICT tools and games	54 (48.18)	3116 (71.01)	57.7 (18.9)
Professional practice	50 (44.64)	576 (13.13)	11.5 (5.7)

^a^ICT: information and communication technology.

##### i-Sharing

A total of 25 discussion threads with 59 posts, including all the replies and follow-up comments, were posted in the i-Sharing forum from July 1, 2019, to July 31, 2020. Approximately 75% (9/12) of NGOs created at least one post, with a median of 2.5 posts. Table 4 shows which ICT tools and their uses had the highest average number of posts per thread, followed by project information.

Table 5 summarizes the number of activities in i-Training, i-Learning, and i-Sharing, as well as the number of project target deliverables. The number of activities of each platform component exceeded the targets by 190% to 3224%.

**Table 4:** Number of discussion threads and posts in the i-Sharing forum.

Category	Threads (N=25), n (%)	Posts (N=59), n (%)	Posts per thread in each category, mean (SD)
ICT^a^ tools and use	8 (32)	41 (70)	5.1 (2.30)
Project information	8 (32)	9 (15)	1.1 (0.35)
Professional practice	9 (36)	9 (15)	1.0 (0)

^a^ICT: information and communication technology.

**Table 5:** Target deliverables and output activities for i-Training, i-Learning, and i-Sharing.

Component	Target deliverables, N	Output activities, N	Target (%)
i-Training (module completions)	141	511	362.4
i-Learning (page views)	132	4388	3324.2
i-Sharing (posts)	31	59	190.3

##### Reactions to i-TLS

Satisfaction with i-TLS on a scale of 0 to 10 showed a median of 5 (IQR 5-7). Table 6 shows a significant difference in satisfaction according to the frequency of use (*P*<.001). Frequent and occasional users showed significantly higher satisfaction than light users, with moderate to large effect sizes (Cohen *d*=0.68-0.80). However, we found no differences between frequent and occasional users. In addition, 74.4% (125/168) of respondents would recommend i-TLS to their colleagues.

In individual phone interviews, the respondents reported that the typical busy schedule of family services made it difficult for them to find resources and learn. i-TLS helped overcome barriers in traditional face-to-face settings, in particular during the COVID-19 pandemic when almost all communication with others shifted to the web. Social workers highlighted the benefits of web-based learning with increased access to resources and reach to colleagues, which provided a good way of continuing development and maintaining service:

I think it is good because it’s accessible to us when we need the resources. It is much easier on a web-based platform, where I could learn anywhere at any time...As you know, we always have a busy schedule in service. With i-TLS, we can revisit the webinars or other resources at our convenience.

Frontline worker, male, 7-8 years of experience

Training and learning that are related to ICT are really rare in social work...except your project. So I think it is quite nice to have many different ICT resources available on a web-based platform. You know, the COVID-19 situation has forced us to adapt to online learning and service.

Supervisor, male, 9-15 years of experience

Family services are very busy...especially when the pandemic doesn’t always allow us to meet face-to-face. It is hard for us to keep everyone updated and teach colleagues how to use ICT tools for program activities as we seldom meet each other. The platform allows us to learn and read through the training resources at our own pace.

Frontline worker, female, 7-8 years of experience

**Table 6:** Survey respondents’ satisfaction with i-TLS and change in knowledge, self-efficacy, and behaviors in applying information and communication technology in family services (N=168).

Variable			Platform users^a^, mean (SD)						Platform users^b^, Cohen *d*						Platform users^c^, *P* value				
			Light users (n=76)		Occasional users (n=79)		Frequent users (n=13)		Occasional vs light users		Frequent vs light users		Frequent vs occasional users		Occasional vs light users		Frequent vs light users		Frequent vs occasional users
**Reactions to i-TLS^d,e^**																			
	Satisfaction	4.99 (1.54)		6.15 (1.34)		6.31 (2.29)		0.80		0.68		0.09		<.001		.01		.94	
**Impact on learning^f^**																			
	Change in knowledge	4.09 (1.74)		5.61 (1.30)		5.84 (1.34)		0.99		1.13		0.17		<.001		<.001		.86	
	Change in self-efficacy	3.96 (1.77)		5.48 (1.29)		5.23 (1.92)		0.98		0.69		0.15		<.001		.02		.86	
**Impact on behavior^g^**																			
	Knowledge application	1.91 (1.40)		4.59 (1.06)		6.46 (1.33)		2.15		3.33		1.55		<.001		<.001		<.001	
	Knowledge sharing	1.80 (1.54)		4.65 (1.31)		5.46 (2.44)		1.99		1.79		0.41		<.001		.001		.18	

^a^*P* value for differences between innovative web-based training, learning, and sharing platform users (3 groups) <.001 in all cases.

^b^Effect size (Cohen *d*): small=0.20, medium=0.50, and large=0.80.

^c^*P* value for the difference between innovative web-based training, learning, and sharing platform users (2 groups).

^d^i-TLS: innovative web-based training, learning, and sharing platform.

^e^11-point Likert scale ranging from 0 to 10 (0=very dissatisfied, 3=dissatisfied, 5=half, 7=satisfied, and 10=very satisfied).

^f^11-point Likert scale ranging from 0 to 10 (0=not at all, 3=slightly increased, 5=moderately increased, 7=highly increased, and 10=very highly increased).

^g^11-point Likert scale ranging from 0 to 10 (0=not at all, 3=seldom, 5=sometimes, 7=almost every day, and 10=every day).

### Impact

#### Impact on Learning

Table 6 shows significant differences in the change in knowledge and self-efficacy in applying ICT to family services according to the frequency of use (*P*<.001). Frequent and occasional users showed a significantly greater increase in knowledge and self-efficacy than light users, with moderate to large effect sizes (Cohen *d*=0.69-1.13). However, we found no differences between frequent and occasional users.

In individual phone interviews, respondents appreciated that the learning resources were easy to understand, which facilitated memorization and application in their daily practice. The web-based shared resources were easy to retrieve and helped reinforce their ICT skills in program implementation:

Many of our services are changing from offline to online now. Those video tutorials on ICT tools really help us a lot, as our colleagues are not active ICT users and do not know how to use them. The videos teach us step-by-step and we know how to use Zoom and other tools to conduct online programs now.

Frontline worker, female, 9-15 years of experience

The recommended ICT tools and other resources on the platform are informative and easy to use. They help strengthen our ICT skills and stimulate ideas in online program implementation. They also help us maintain online services during the pandemic.

Frontline worker, male, 3 years of experience

The game demonstration videos helped remind me of things I had done and learned from other NGO partners during the project launch. Sometimes, I might have forgotten and am not sure how it [the game] works, so it is nice to have that documentation...I will follow the steps and do it again in my program.

Frontline worker, male, 9-15 years of experience

Respondents also believed that i-TLS facilitated knowledge transfer within their professional practice. They especially enjoyed learning from the experiences of workers from other centers. Sharing information in i-TLS boosted their confidence and was highly applicable to their service, which benefited everyone by filling the gaps between knowledge and practice. It also built an environment of colearning:

I guess it [i-TLS] is like a learning hub...we are learning together. We have more confidence in applying ICT after reading others’ experience sharing. Their sharing provides us concrete ways of implementation, which also helps stimulate new ideas in practice. It is nice to learn from others.

Frontline worker, male, 5-6 years of experience

We like something practical...my colleagues and I like skimming through the examples of the program plans and designs from other workers. They are very good ideas for reference. It helps facilitate our learning through knowing how to do it in practice.

Frontline worker, female, 7-8 years of experience

Many social workers from other family centers shared their experiences in applying ICT to engage vulnerable families. I learned a lot of practical skills from their experiences and was able to apply them to my work.

Frontline worker, male, 1 year of experience

#### Impact on Behavior

Table 6 shows significant differences in applying and sharing knowledge according to the frequency of use (*P*<.001). Frequent and occasional users showed a significantly greater increase in applying and sharing the acquired knowledge in family services than light users, with large effect sizes (Cohen *d*=1.79-3.33). Frequent users also showed a significantly greater increase in applying the acquired knowledge to family services than occasional users, with a large effect size (Cohen *d*=1.55). However, we found no differences between frequent and occasional users in knowledge sharing.

In individual phone interviews, respondents had mixed views on whether ICT tools should be used or how they should be supported in family services. In general, respondents felt that ICT offered many benefits, and they saw the potential use of ICT in family services after knowing more about ICT and its related resources. They became more open to ICT and increased ICT use in family services:

It was challenging to my colleagues when it started, especially to those who were not active ICT users...But we see the advantages of them and more of our colleagues are using ICT in service now. The materials on the platform are good sources for us to start off.

Supervisor, female, 9-15 years of experience

I see my colleagues changing their attitude toward ICT use after using the platform. In the past, they were very reluctant to apply ICT tools. Most of them preferred face-to-face as their usual practice. However, they are more willing to incorporate ICT tools into their program now. They also take the initiative to explore the ICT tools on i-TLS and use more ICT in their program.

Frontline worker, male, 2 years of experience

However, a few respondents were not very positive. Some remained hesitant about adopting ICT in family services. They reported that barriers included the absence of human quality and emotional bonding during counseling. How to navigate the electronic environment to effectively apply learning to an in-person practice setting remains a challenge to some social workers:

Counseling is somewhat relational that involves human interactions and emotional bonding while ICT is more informative...I think counseling cannot be replaced by ICT completely. We need to think about how to flexibly incorporate ICT into this ever-changing environment and to facilitate current service.

Supervisor, male, 9-15 years of experience

ICT might be useful, but it does not play a large role in motivating service users and enhancing the counseling process. Face-to-face interaction is still a priority in our service, especially in casework.

Frontline worker, female, 1 year of experience

Service users’ ability to use ICT could also affect social workers’ attitudes and use of ICT tools during program activities. Indeed, as the respondents reported, not all service users were information technology–literate and had access to electronic devices, which was essential for ICT use. Therefore, social workers usually preferred adopting simple or usual ways in their practice to meet the needs of service users:

So...the way things work usually for our service users is very much sort of pen and paper. The outcomes can be very different from using a laptop or smartphone for many of our service users. We need to think carefully about what devices or ICT tools suit our service users...we usually go back to the basics or stick to the old practice.

Frontline worker, male, 3 years of experience

## Discussion

### Principal Findings

We have reported the development and evaluation of a web-based platform aimed at supporting social workers’ learning during the early stage of digital transformation in social work family practice. The results provided insights into the acceptability and impact of an innovative learning platform with 3 different components and learning approaches. Overall, we enrolled 313 social workers, with around 80% (249/313) of active users at 13 months. The demographic characteristics of the respondents revealed similar trends to those in the statistics report from the Hong Kong Social Workers Registration Board [26]. Although the pandemic might have led to more ICT use and targets being exceeded, the continuous increase in the number of users and platform activity showed that the platform was acceptable and useful in its first year but with room for further improvements and increase in use. Users were satisfied with the platform, and more frequent users showed increased knowledge, self-efficacy, and use of ICT in services compared with light users. Through focus groups and individual phone interviews, we found nuances in motivation and behavior change, which gave us a better understanding of how this platform influenced the learning of social workers and their clinical practice.

Users in this study were positive about the potential use of i-TLS in supporting continuous learning. They felt that i-TLS could increase accessibility to learning in a way that might not be possible in traditional face-to-face settings. Some admitted that they felt unease in finding time for learning amid the busy professional duties and time pressures they faced in clinical settings. These findings support previous assertions by clinicians and researchers that the asynchronous and hybrid nature of web-based learning allows for more learning opportunities [12] and provides more access to a wide range of information independent of space and time [27]. These findings are also consistent with a British sample of social workers in a web-based learning pilot study [28], who also felt that web-based platforms could increase flexibility and autonomy for learning.

Importantly, our data suggest that i-TLS could increase support by reaching more social workers across centers at the point of need, particularly during the outbreak of COVID-19. Given the primary face-to-face nature of social work practice, keeping social workers engaged in such a digital environment is challenging. However, the rapid transformation and need for digitalization in this specific situation necessitated the use of web-based learning and new communication modes to maintain the quality of professional development and service delivery. Training on data best practices and video tutorials on web-based communication tools and creative platforms such as Zoom videoconference, YouTube, and Prezi have become the most popular materials among social workers. Social workers across centers were more willing to use and share information over time as they learned more about the platform’s capabilities. Before launching i-TLS, the exchange of information, ideas, and knowledge on ICT use across centers was limited, with little peer collaboration. Although books [29,30] and social work professional bodies such as the National Association of Social Workers and the Association of Social Work Boards [31] have recommended ways of ICT use and set new standards regarding social workers’ ability to use ICT in practice earlier, factors such as limited access to ICT-related training and resources mean that many social workers and service users may not be able to receive timely access to the support they need during this difficult time. It is expected that the demand for and use of web-based learning will continue to increase.

We found that frequent users showed a higher score in knowledge and self-efficacy than light users. Users expressed that i-TLS encouraged collaborative learning through sharing of knowledge and experiences in the sense of a learning community. The adopted collaborative learning approach created a peer-to-peer environment that could benefit users’ learning through learning from others [32]. As such, knowledge acquisition occurred not only via self-experiences but also indirectly through the experiences of others. Successful presentations with applause and appreciation by peers created a sense of success and positive feeling regarding their learning experiences, which boosted the self-efficacy of both presenters and attendees. According to the self-efficacy theory [33,34], one’s self-efficacy increases not only through positive personal experiences but also by seeing others succeed. There has been an increasing awareness and understanding of learning from others within higher education [35,36] and various professions that require clinical practice [32,37,38]. However, this learning approach has not been well-described in the social work learning literature. The cocreating feature of our i-TLS, which fosters the sharing of knowledge and experiences, was identified as a strength by the users.

Despite concerns about human interaction and some practical barriers inherent in ICT tools, the attitude of the participating social workers toward the use of ICT in practice was largely positive and much more open after using i-TLS, especially under the challenges of social distancing measures amid the pandemic. We found that frequent users, who showed higher perceived knowledge and self-efficacy, used ICT in services more frequently. However, some social workers remained hesitant to fully embrace technology and expressed concerns about its practical use, especially in traditional in-person services such as counseling, although they were aware of the benefits of incorporating ICT into their practice. Considering the principles of social work ethics, which value the central importance of human relationships [39], social workers may see ICT as only a supplementary tool and less important. Therefore, those who emphasize the development of in-person relationships may remain struggling to incorporate ICT into practice to maintain emotional bonding with clients.

By contrast, this suggests that the perception of users toward ICT is also a key factor influencing the adoption of ICT. These findings are largely supported by the theory of planned behavior, with one’s belief, perceived behavioral control, and intention predicting a behavior [40]. A review of ICT use in Norwegian social work practice [41] also suggested that the perception of new ICT solutions may affect how far and well they spread and evolve according to the diffusion of innovation theory by Roger [42]. Knowing the benefits and understanding alone does not ensure widespread adoption; rather, it requires changes in the values, beliefs, and needs of the adopter. The reluctance of some social workers to use ICT suggests that the information currently available does not adequately address their concerns. With the increasing demand for web-based services and the propensity to seek help on the web [43], it is essential for social workers to master ICT practices from scheduling appointments to program implementation, therapeutic discussions, and counseling. To enhance social workers’ professional development, experienced practitioners in the field indeed play an important role in wisdom sharing and providing a clearer understanding of building effective web-based relationships. Their sharing may help lay the foundation for this new trend in using ICT to achieve the desired values of social work. Future educators and researchers should put more effort into ensuring that the ICT training content promotes both professional and technical improvement and enhancement of services.

### Limitations

This was the first evaluation of i-TLS approximately 1 year after its official launch [13]. Our study had some limitations. The survey sample size and response rate were not great. This is similar to those reported in the literature [44], which showed a relatively lower participation rate of social workers on a learning and sharing platform than other service providers across care centers. Owing to the busy schedule in family services, especially challenges from the pandemic, many social workers were not able to set aside the time to use the platform or complete the survey questionnaire. In addition, the culture of some agencies relied on the coordinator to collate information, which further limited social workers’ self-learning and individual access to the platform. Before the SMART Family-Link Project, most participating social workers were in the same form of training or work environment with very limited exposure to ICT in their centers. i-TLS was their first web-based learning platform, and some would be slow to join and adopt it. Moreover, we did not have objective data on knowledge and behaviors, and self-reported data might be subject to social desirability bias. Objective assessments in future research are warranted. Consequently, our results might not be generalizable to all social workers and other social welfare services. Nevertheless, these limitations should not detract from our findings, which suggest that the participating social workers were eventually well-versed in the benefits of and barriers to ICT use in family services.

### Recommendations

The feedback from i-TLS users revealed some important elements that researchers and technical developers need to consider when developing web-based learning platforms, whereas the results of this evaluation also underscored the need for further enhancement of the platform to help social workers learn. Additional promotion and engagement are required to further increase the use and ensure that social workers receive sufficient support to access web-based resources. Attempts are continuously being made to improve the i-TLS platform’s layout and functionality. A revamped version of i-TLS was officially launched on June 28, 2021, and access to the site can be provided upon request. From the results of this study, literature reviews, and social work feedback, we propose several recommendations (Textbox 2).

**Recommendations for the development of web-based learning platforms.**

**Recommendations for the web-based learning format**

- The accessibility, convenience, and simplicity that came from the platform and digital resources were valued.
- Videos, including animations, module recordings, and demonstration clips, were the preferred types of e-learning resources among participating social workers, which should be considered in future web-based learning design in social work curricula.
- Various formats of web-based learning and their combinations, including videos, animated clips, text documents, or links to external web-based resources, as opposed to a single delivery mode, may address a diversity of needs and increase the users’ engagement. Further research on the effectiveness of using web-based learning and training by integrating various formats of learning is needed.
- More graphics, animated text documents, and illustrations should be provided to increase user engagement and adherence. As suggested by Keller and Suzuki [45] in a motivation model of instructional design, the learning process begins by providing knowledge and information using multimedia such as animation videos, text, and illustrations to gain and sustain attention.

**Recommendations for professional development**

- Training and resources that facilitated applicability in services bridged the gaps between knowledge and practice, which were perceived as important to social workers’ professional development.
- Sharing of experiences supported social workers’ learning process and created valuable content that maximized their experiential learning.
- Documentation of procedures helped memorization and reinforced social workers’ skills in practice.
- The learning together environment provided a potential means by which social workers could be inspired and then generate more creative ideas that benefited their services. A cocreating nature was identified as a strength by users.
- A diverse collection of learning items should be included to fit the needs of social workers with various backgrounds and interests to increase learning engagement.
- Key leaders in social work and health-related fields sharing valuable resources and feedback may play an essential role in social workers’ professional development. Leaders are expected to play active roles in initiating chat topics and sharing valuable insights, which could draw social workers’ attention and facilitate more sharing and participation on the platform.

### Conclusions

We have shown that the newly developed learning platform for family service social workers is acceptable and effective, as reflected in the substantial growth in platform use and increased knowledge, self-efficacy, and ICT use in family services. Social workers identified some key elements through which i-TLS was perceived to optimize their learning, including enabling access to promote self-directed and collaborative learning and encouraging sharing of experiences within their practice. i-TLS can foster unique collaborations across boundaries by allowing social workers from different service centers to interact and share equally in a colearning environment without time and space constraints. Our results should be a useful reference for researchers, health and social care professionals, and administrators considering developing a web-based learning platform for continuous professional development. Further research on the development and enhancement of web-based platforms to promote and expand the participation and capacity building of social workers and other related professionals is warranted.

## Appendix

Multimedia Appendix 1 Screenshots of the innovative web-based training, learning, and sharing platform (i-TLS) displaying the home page and the interface of the three individual components (training, learning, and sharing).

## Abbreviations

HKU: University of Hong Kong
ICT: information and communication technology
i-TLS: innovative web-based training, learning, and sharing platform
NGO: nongovernmental organization

## References

[1] Mishna F, Bogo M, Root J, Sawyer J, Khoury-Kassabri M (2012). “it just crept in”: the digital age and implications for social work practice. Clin Soc Work J.

[2] Baker S, Warburton J, Hodgkin S, Pascal J (2018). The new informational paradigm: developing practice-led approaches to the use of mobile ICT in social work. Brit J Soc Work.

[3] Reamer FG (2013). Social work in a digital age: ethical and risk management challenges. Soc Work.

[4] Baker S, Warburton J, Hodgkin S, Pascal J (2014). Reimagining the relationship between social work and information communication technology in the network society. Aus Social Work.

[5] Chan C, Holosko MJ (2015). A review of information and communication technology enhanced social work interventions. Res Social Work Pract.

[6] (2018). Harness technology for social good. Grand Challenges for Social Work and Society.

[7] López Peláez A, Pérez García R, Aguilar-Tablada Massó M (2017). e-Social work: building a new field of specialization in social work?. Eur J Social Work.

[8] Ross DA, National Neuroscience Curriculum Initiative “Quarantine Curriculum” Committee (2020). Creating a "Quarantine curriculum" to enhance teaching and learning during the COVID-19 pandemic. Acad Med.

[9] Iancu AM, Kemp MT, Alam HB (2020). Unmuting medical students' education: utilizing telemedicine during the COVID-19 pandemic and beyond. J Med Internet Res.

[10] Mishna F, Milne E, Bogo M, Pereira L (2020). Responding to COVID-19: new trends in social workers' use of information and communication technology. Clin Soc Work J.

[11] Aafjes-van Doorn K, Békés V, Prout T (2020). Grappling with our therapeutic relationship and professional self-doubt during COVID-19: will we use video therapy again?. Counsell Psychol Q.

[12] Xenakis N (2018). Creating a professional development platform to transform social work clinical practice in health care. Soc Work Health Care.

[13] Sit S, Lai A, Kwok T, Wong H-W, Wong Y-L, Lam EY, Chan JY, Kong FS, Cham K, Ng CK, Yip T, Tsui TS, Wong C-M, Wong BC, Tang W-Y, Yam P-W, Chui M, Wan A, Kwok Y-K, Lam T-H (2020). Process evaluation and experience sharing on utilizing information communication technologies and digital games in a large community family health event: Hong Kong jockey club SMART family-link project. Front Public Health.

[14] Pope C, Mays N (1995). Reaching the parts other methods cannot reach: an introduction to qualitative methods in health and health services research. BMJ.

[15] Garrison DR (1997). Self-directed learning: toward a comprehensive model. Adult Educ Q.

[16] Wenger E (1998). Communities of Practice Learning, Meaning, and Identity.

[17] Durning S, Artino A (2011). Situativity theory: a perspective on how participants and the environment can interact: AMEE Guide no. 52. Medical Teacher.

[18] Sweller J (1988). Cognitive load during problem-solving effects on learning. Cognit Sci.

[19] Sweller J, van Merriënboer JJ, Paas F (1998). Cognitive architecture and instructional design. Educ Psychol Rev.

[20] van Merriënboer JJ, Sweller J (2005). Cognitive load theory and complex learning: recent developments and future directions. Educ Psychol Rev.

[21] Szulewski A, Howes D, van Merriënboer JJ, Sweller J (2021). From theory to practice: the application of cognitive load theory to the practice of medicine. Acad Med.

[22] Chan AK, Botelho MG, Lam OL (2019). Use of learning analytics data in health care-related educational disciplines: systematic review. J Med Internet Res.

[23] Lachenbruch PA, Cohen J (1989). Statistical power analysis for the behavioral sciences (2nd edition). J Am Stat Assoc.

[24] Braun V, Clarke V (2006). Using thematic analysis in psychology. Qual Res Psychol.

[25] Mays N, Pope C (1995). Rigour and qualitative research. BMJ.

[26] Statistics on registered social workers. Social Workers Registration Board.

[27] Kotzer S, Elran Y (2012). Proceedings of the 1st Moodle Research Conference.

[28] Webber M, Currin L, Groves N, Hay D, Fernando N (2010). Social workers e-Learn: evaluation of a pilot post-qualifying e-Learning course in research methods and critical appraisal skills for social workers. Soc Work Educ.

[29] Hill A, Shaw I (2011). Social Work and ICT.

[30] Watling S, Rogers J (2012). Social Work in a Digital Society.

[31] National association of social workers and association of social work boards standards for technology and social work practice. National Association of Social Workers.

[32] Roberts D (2010). Vicarious learning: a review of the literature. Nurse Educ Pract.

[33] Bandura A (1994). Self-efficacy. Encyclopedia of Health and Behavior.

[34] Bandura A (1977). Self-efficacy: toward a unifying theory of behavioral change. Psychol Rev.

[35] Ashworth P (2004). Understanding as the transformation of what is already known. Teach High Educ.

[36] Ellis RA, Calvo R, Levy D, Tan K (2004). Learning through discussions. High Educ Res Dev.

[37] Stegmann K, Pilz F, Siebeck M, Fischer F (2012). Vicarious learning during simulations: is it more effective than hands-on training?. Med Educ.

[38] Meyer M, Marzen-Groller K, Myers S, Busenhart C, Waugh S, Stegenga K (2014). Simulation as a learning experience: perceptions of new RNs. Clin Simulation Nurs.

[39] Read the code of ethics. National Association of Social Workers.

[40] Ajzen I (1991). The theory of planned behavior. Org Behav Human Decision Processes.

[41] Zhu H, Andersen S (2020). ICT-mediated social work practice and innovation: professionals’ experiences in the Norwegian Labour And Welfare Administration. Nordic Social Work Res.

[42] Roger EM (2010). Diffusion of Innovations.

[43] Gupta A, Agrawal A (2012). Internet counselling and psychological services. Soc Sci Int.

[44] McLinden D, Myers S, Seid M, Busch M, Davis D, Murphy J (2019). The learning exchange, a community knowledge commons for learning networks: qualitative evaluation to test acceptability, feasibility, and utility. JMIR Form Res.

[45] Keller J, Suzuki K (2004). Learner motivation and E-learning design: a multinationally validated process. J Educ Media.

