# Supplementary material for: Development and Evaluation of an Innovative Web-Based Training, Learning, and Sharing Platform for Social Workers (Hong Kong Jockey Club SMART Family-Link Project): Mixed Methods Evaluation Study
Source: JMIR Form Res. 2022 Apr 28;6(4):e32894. doi: 10.2196/32894 (PMC9100379; doi:10.2196/32894)

Multimedia Appendix 1

Screenshots of the i-TLS platform displaying the homepage and the 3 individual components (Training, Learning, and Sharing) interface.

Figure S1. The homepage of i-TLS


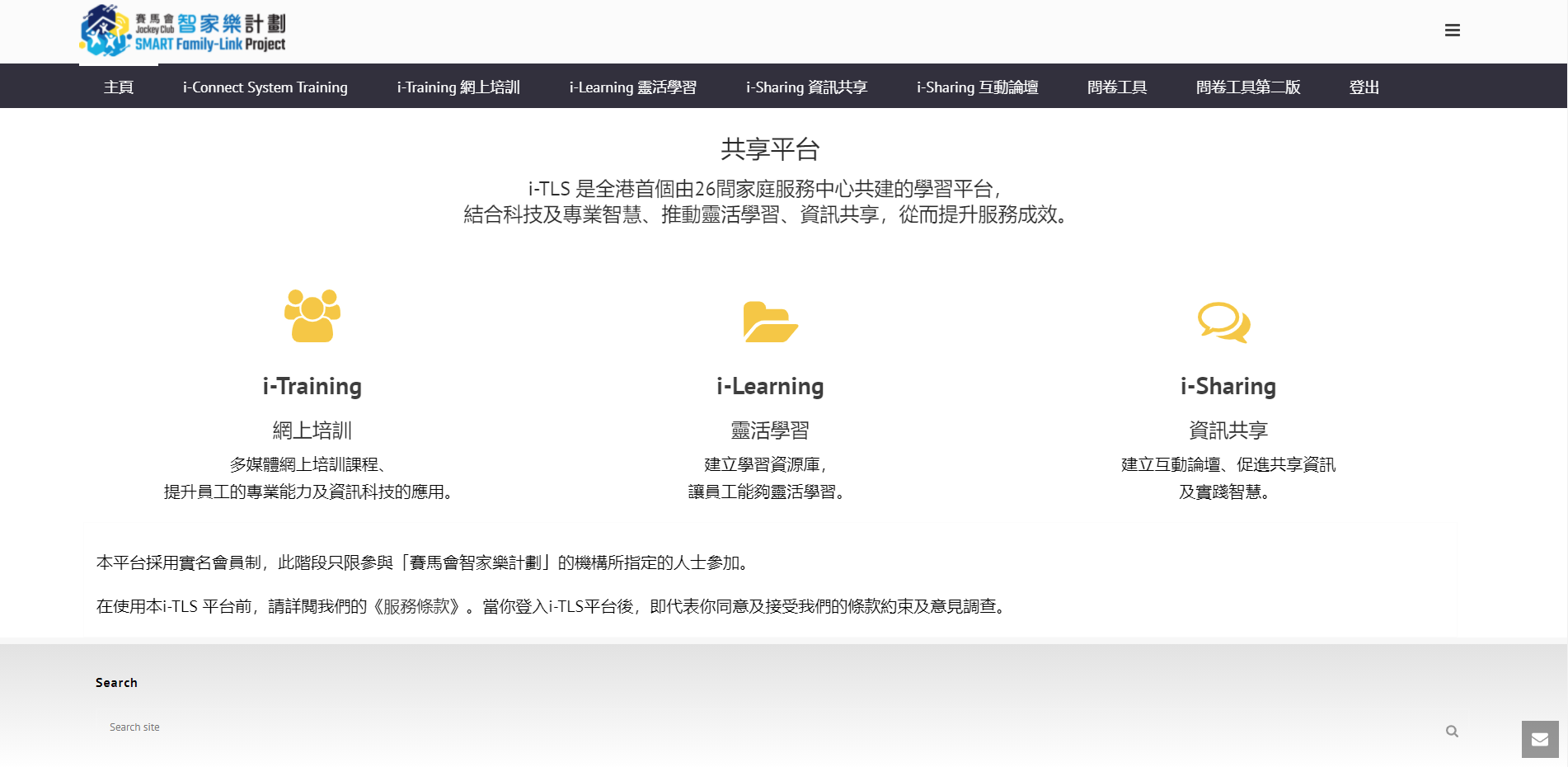


Figure S2. The content of the i-Training modules.


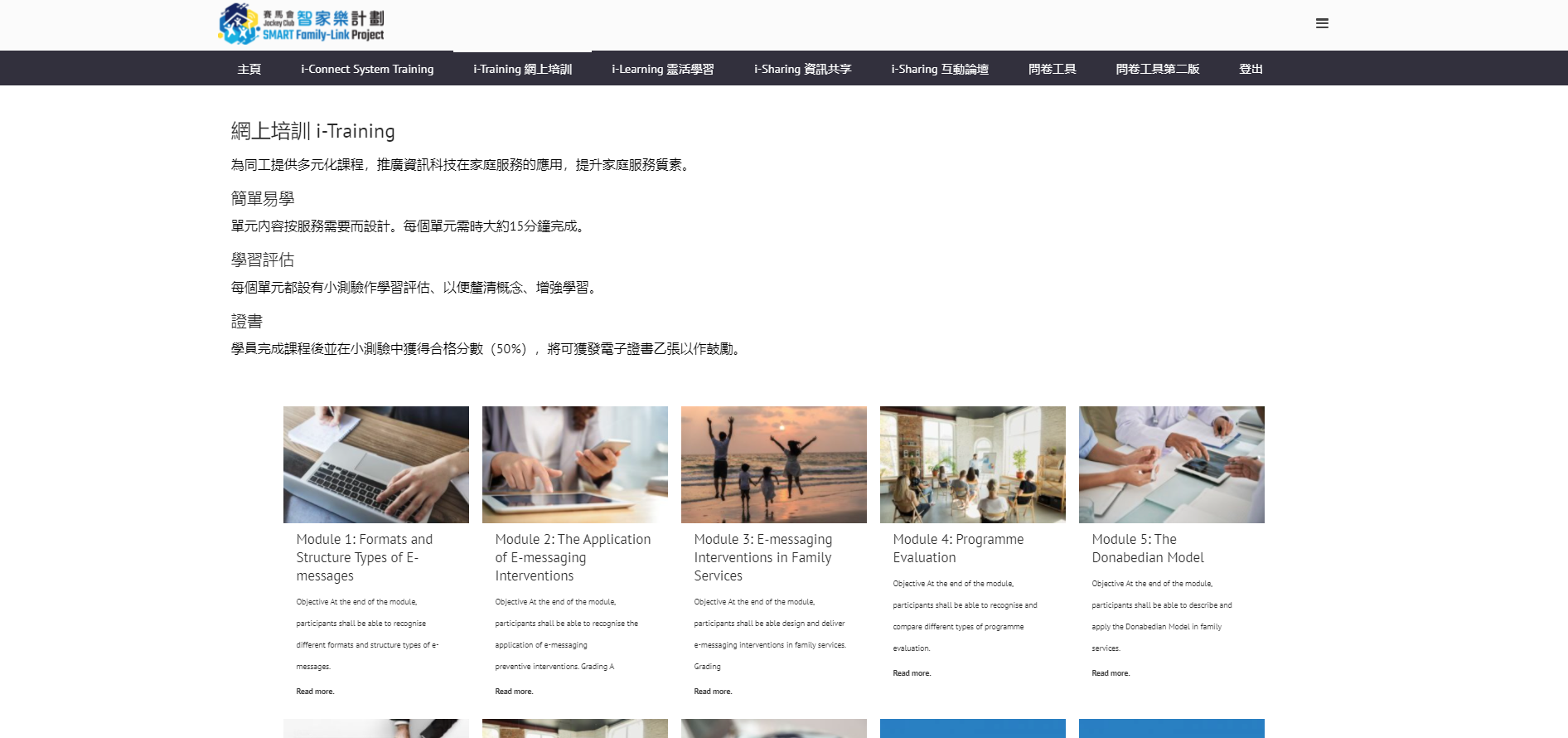


Figure S3. An example of the i-Learning resources.


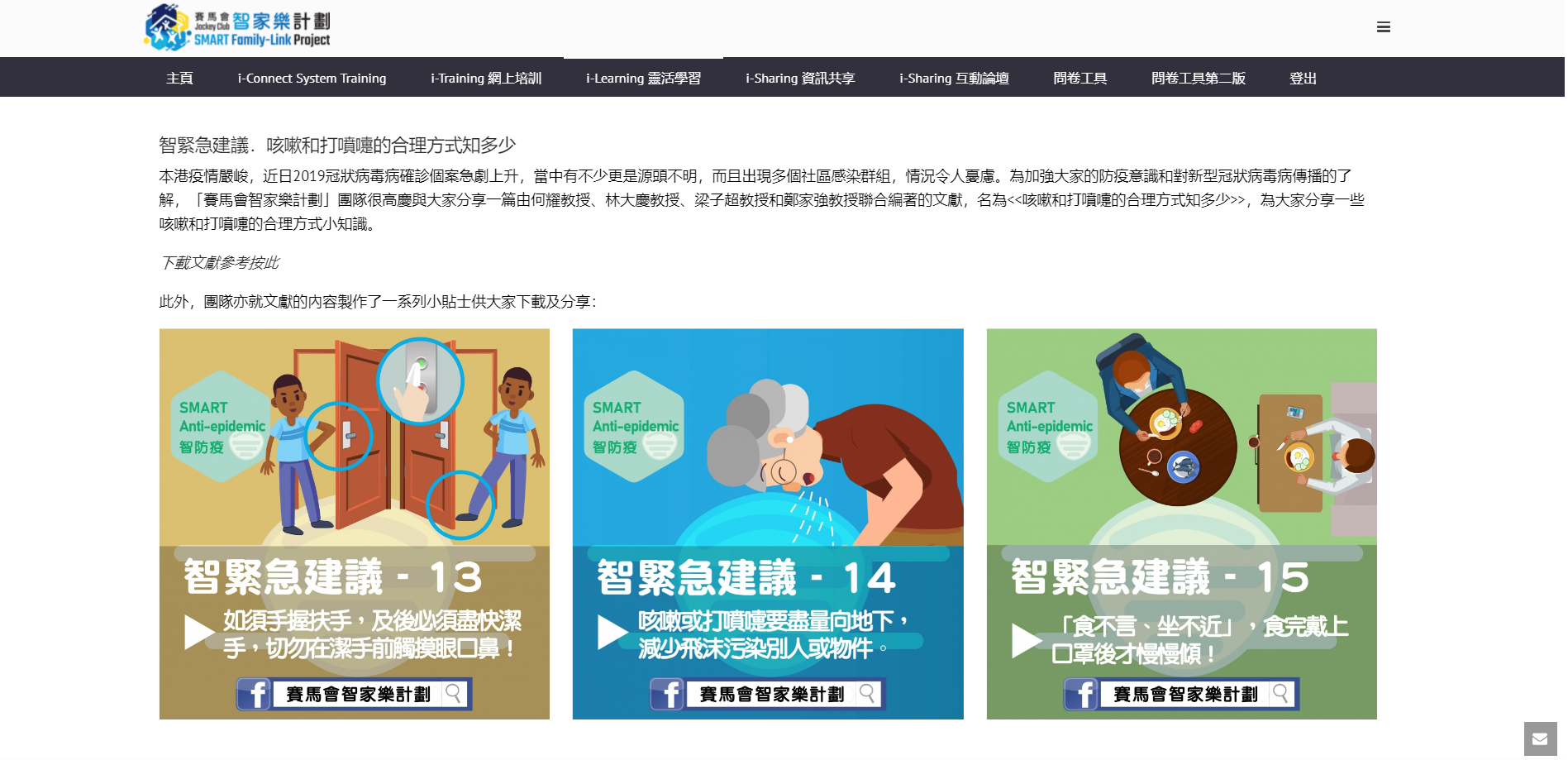


Figure S4. The area of postings on i-Sharing forum.


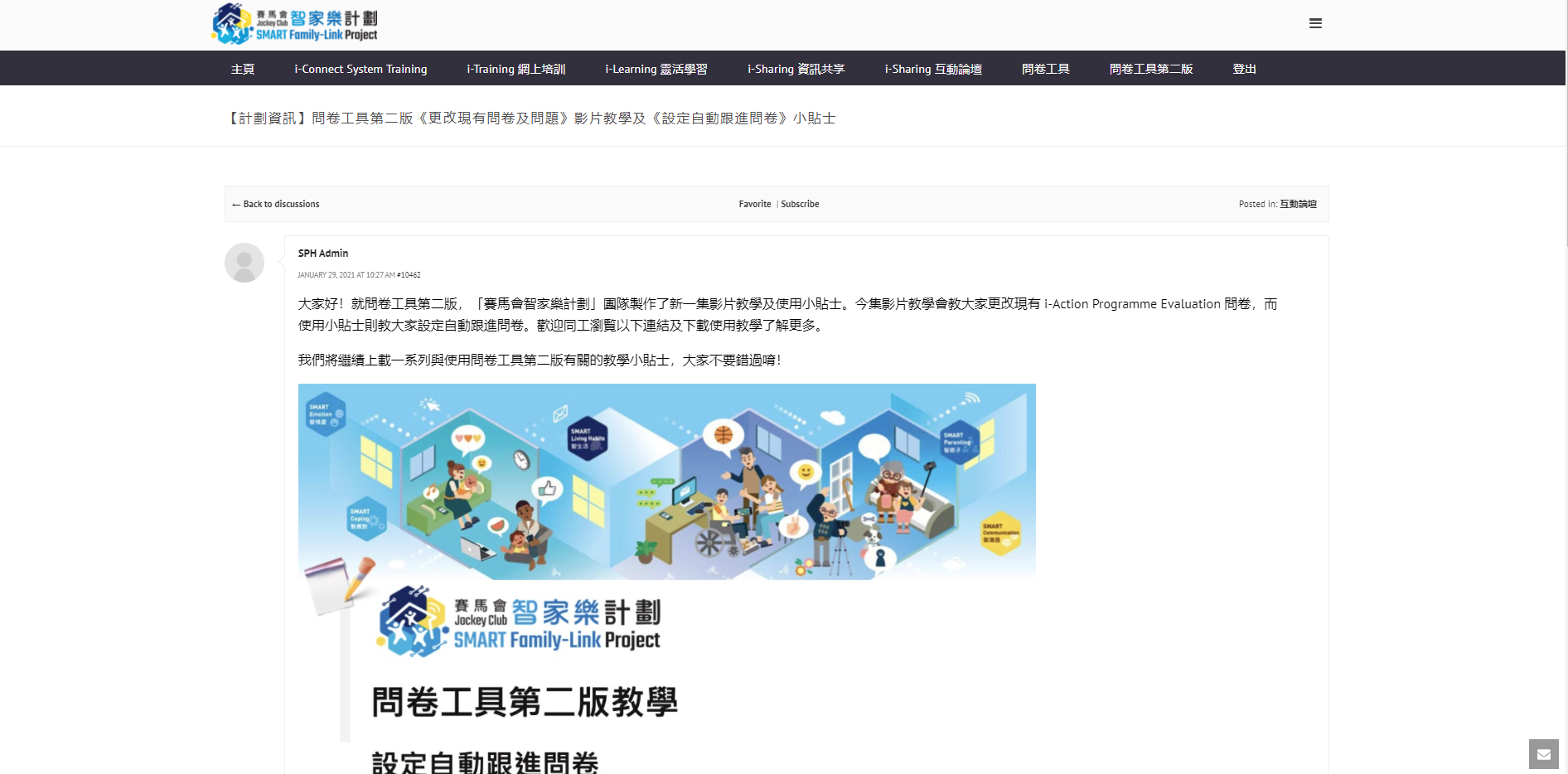

Supplement: Multimedia Appendix 1 [file formative_v6i4e32894_app1.docx]
